# Osimertinib acquired resistance among patients with EGFR-mutated NSCLC: from molecular mechanisms to clinical therapeutic strategies

**DOI:** 10.20517/cdr.2025.140

**Published:** 2025-12-02

**Authors:** Ren Wang, Yuanhang Chen, Liping Li, Lun Zhang, Sheng Zhang

**Affiliations:** ^1^Affiliated Cancer Hospital and Institute, Guangzhou Medical University, Guangzhou 511436, Guangdong, China.; ^2^Institute of Biomedical Engineering, Kunming Medical University, Kunming 650500, Yunnan, China.; ^3^Guangzhou Institutes of Biomedicine and Health, Chinese Academy of Sciences, Guangzhou 510530, Guangdong, China.; ^4^Department of Gastroenterology, Guangdong Provincial Second Hospital of Traditional Chinese Medicine/Guangdong Provincial Engineering Technology Research Institute of Traditional Chinese Medicine, Guangzhou 510095, Guangdong, China.; ^#^These authors contributed equally to this work.

**Keywords:** NSCLC, osimertinib resistance, molecular mechanisms, clinical therapeutic strategies

## Abstract

Non-small-cell lung cancer (NSCLC) remains the leading cause of global cancer-related mortality. NSCLC patients with epidermal growth factor receptor (EGFR) mutations benefit substantially from treatment with EGFR tyrosine kinase inhibitors, particularly osimertinib. Although recent clinical trials have established osimertinib as effective treatment across many stages of EGFR-mutant NSCLC, the inevitable emergence of acquired resistance poses a major therapeutic challenge despite the substantial clinical benefit. Understanding the mechanisms of osimertinib acquired resistance is urgently needed to identify effective strategies to overcome it. Resistance to osimertinib including on-target mechanisms such as novel EGFR secondary mutation, off-target mechanisms such as mesenchymal-epithelial transition or human EGFR 2 amplification, mutations in downstream signaling molecules, and oncogenic fusions, and the Histological transformations (such as epithelial-mesenchymal transition, squamous cell carcinoma, or small cell lung cancer) have been well described. This review summarizes the mechanisms and clinical significance of osimertinib-acquired resistance in recent years, as well as new clinical treatments. It is expected to provide valuable insights and potential new strategies for the clinical treatment of EGFR-mutated NSCLC patients with osimertinib resistance.

## INTRODUCTION

Non-small-cell lung cancer (NSCLC) is the most prevalent and lethal form of lung malignancy, exerting a significant global impact^[1-3]^. The epidermal growth factor receptor (EGFR), a transmembrane receptor tyrosine kinase (RTK), is frequently mutated in NSCLC and represents the predominant driver mutation in this malignancy^[4,5]^. The prevalence of EGFR mutation rate is notably high among Asian patients with NSCLC, reaching 30%-50%^[6-8]^. The two most common activating mutations-exon 19 deletions (Ex19del) and the L858R point mutation in exon 21-collectively account for approximately 90% of all EGFR mutations^[9]^. These alterations of EGFR can serve as the therapeutic target for EGFR-tyrosine kinase inhibitors (TKIs)^[10]^.

Patients with EGFR-mutant NSCLC derive significant clinical benefit from EGFR-TKI therapy. Osimertinib, a third-generation EGFR-TKI, has become the global standard care for the first-line treatment for the locally advanced EGFR-mutant NSCLC^[11]^. It effectively overcomes threonine^790^-to-methionine^790^ (T790M)-mediated resistance to first- or second-generation EGFR-TKIs. In the phase III AURA3 trial (NCT02151981), osimertinib was established as the gold standard for second-line treatment in NSCLC patients with EGFR T790M mutation, outperforming chemotherapy in efficacy and safety^[12,13]^. Notably, osimertinib has also shown significant improvements in overall survival (OS), disease-free survival (DFS), and objective response rate (ORR) in NSCLC patients without or with undetermined EGFR T790M mutation status. Furthermore, it is effective in treating brain metastases or leptomeningeal metastases, regardless of T790M mutation status^[14,15]^. Moreover, the FLAURA trial (NCT02296125) has revolutionized the treatment paradigm by demonstrating that osimertinib is superior to the first-generation EGFR-TKIs (Gefitinib/Erlotinib) as first-line treatment for patients with metastatic NSCLC harboring EGFR Ex19del or L858R mutation. Osimertinib showed clear superiority to gefitinib or erlotinib, with a nearly twofold improvement in progression-free survival (PFS) and a proven OS benefit^[16,17]^. Given its status as the standard first-line therapy for advanced EGFR-mutant NSCLC, osimertinib is universally endorsed as a priority recommendation by leading global guidelines, including the National Comprehensive Cancer Network (NCCN), European Society for Medical Oncology (ESMO), and Chinese Society of Clinical Oncology (CSCO)^[3,18,19]^. Therefore, osimertinib monotherapy or osimertinib-based combination chemotherapy is recommended for patients with advanced EGFR-mutant NSCLC.

In patients with early-stage EGFR-mutant NSCLC, the ADAURA trial (NCT02511106) demonstrated that adjuvant osimertinib significantly improved both OS and PFS following complete resection^[20,21]^. Additionally, the LAURA trial (NCT03521154) showed that in patients with unresectable stage III EGFR-mutant NSCLC who had received definitive chemoradiotherapy, osimertinib significantly prolonged PFS compared with placebo^[22,23]^. Furthermore, Phase II data from the NEOS trial (ChiCTR1800016948), along with smaller studies/real-world datasets, has confirmed that neoadjuvant osimertinib is feasible, effective, and safe in patients with operable EGFR-mutant early-stage (IIA-IIIB) NSCLC^[24,25]^. Notably, a single-arm study reported that neoadjuvant osimertinib was well tolerated, but failed to significantly improve the major pathological response (MPR) rate^[26]^. However, the global multicenter, phase III NeoADAURA trial (NCT04351555) demonstrated that osimertinib monotherapy or combination with chemotherapy significantly improved the MPR rate and increased the opportunity for R0 resection in patients with resectable EGFR-mutant NSCLC^[27]^. Similarly, the NORA trial (NCT04816838) confirmed that neoadjuvant osimertinib plus chemotherapy significantly improved the MPR compared with chemotherapy alone in patients with operable, stage IA-IIIB EGFR-mutant NSCLC^[28,29]^. To further improve treatment outcomes, FLAURA2 (NCT04035486) and MARIPOSA-2 (NCT04988295) have initiated a new phase of osimertinib-based combination treatment strategies for first-line treatment of EGFR-mutant NSCLC^[30,31]^. In patients with EGFR-mutant NSCLC, osimertinib is now integrated across the disease continuum - as adjuvant therapy after complete resection, neoadjuvant therapy prior to surgery, and consolidative therapy following definitive chemoradiotherapy for unresectable locally advanced disease.

Although osimertinib benefits patients with EGFR-mutant NSCLC across early-stage, locally advanced, and metastatic settings, disease progression driven by acquired resistance remains inevitable. Recurrence during osimertinib therapy, adjuvant osimertinib, or consolidative osimertinib after chemoradiotherapy indicates emergence of resistance to osimertinib^[32]^. Thus, osimertinib resistance in EGFR-mutant NSCLC remains a critical unresolved challenge and an active area of research. Overcoming this resistance is imperative. Here, by reviewing the mechanisms underlying osimertinib resistance and the latest therapeutic strategies to manage it, we aim to unveil innovative insights and directions to surmount this clinical barrier.

## ACQUIRED MECHANISM OF OSIMERTINIB RESISTANCE

Elucidating the mechanisms of osimertinib resistance is essential for guiding precision therapy in patients who progress on osimertinib. At relapse, re-biopsy to obtain fresh tumor tissue is prioritized; adequate material is obtained in approximately 50% of cases^[33]^. The specimen is then subjected to next-generation sequencing (NGS) to identify emergent somatic genomic alterations, to proteomic analysis and fluorescence *in situ* hybridization (FISH) to detect oncogene amplifications [e.g., mesenchymal-epithelial transition (MET)] or increased protein expression, and to histopathological examination to uncover potential histologic transformation^[34]^. When tissue re-biopsy is not feasible, liquid biopsy is employed as an alternative; high-throughput sequencing of plasma circulating tumor DNA (ctDNA) identifies the acquired genomic alterations underlying resistance^[35]^.

**Figure 1:**
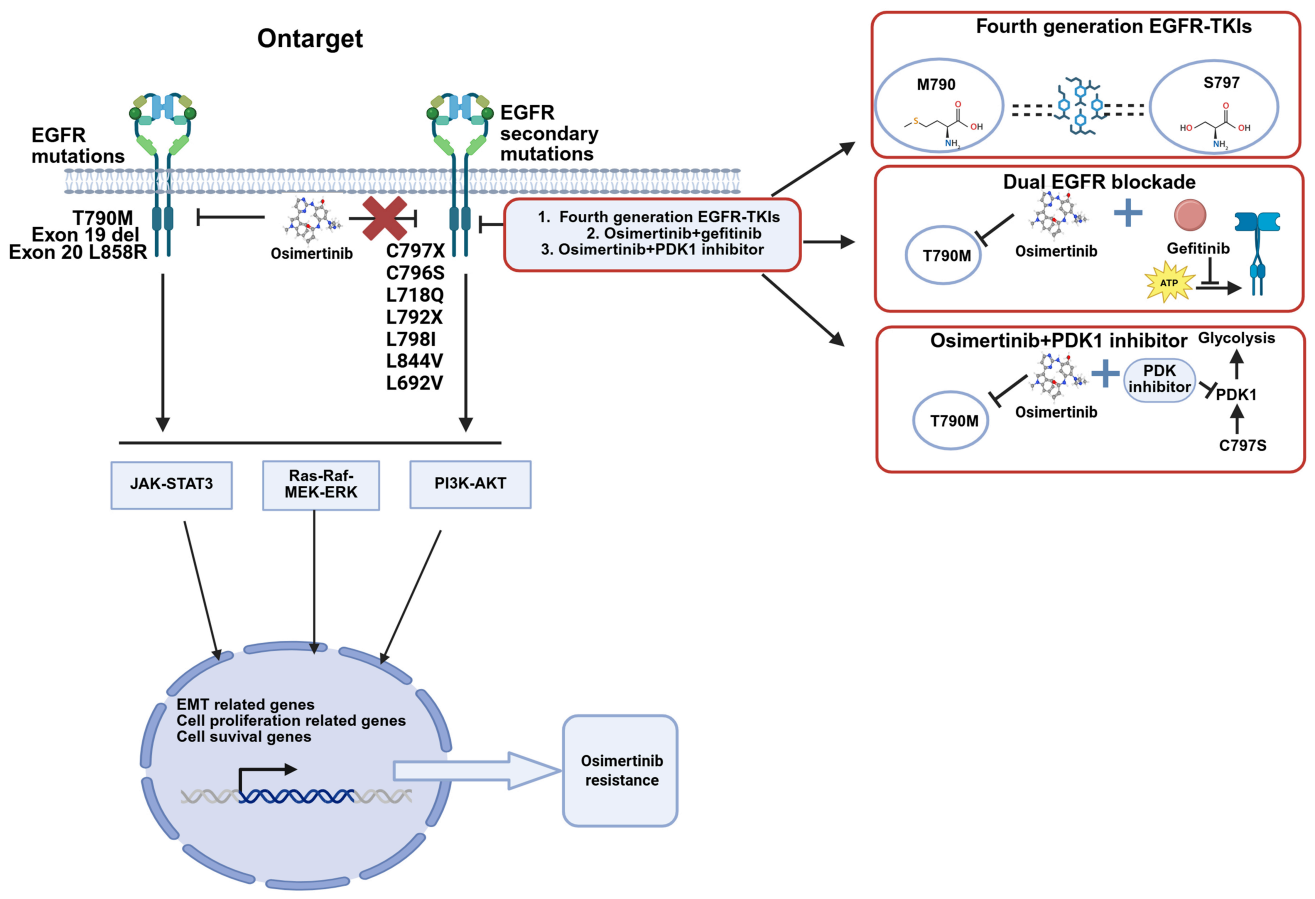
On-target mechanisms of osimertinib-acquired resistance in EGFR-mutated NSCLC and strategies to overcome them, including EGFR secondary mutations and amplification observed in first-line and second-line treatment. Created with BioRender.com (https://BioRender.com/18y6p5u). EGFR: Epidermal growth factor receptor; TKI: tyrosine kinase inhibitor; JAK-STAT3: Janus kinase-signal transducer and activator of transcription 3; Ras-Raf-MEK-ERK: Ras-rapidly accelerated fibrosarcoma-mitogen-activated protein kinase kinase-extracellular signal-regulated kinase; PI3K-AKT: phosphatidylinositol 3-kinase-protein kinase B; EMT: epithelial-mesenchymal transition; PDK1: phosphoinositide-dependent kinase 1; NSCLC: non-small-cell lung cancer; ATP: adenosine triphosphate.

**Figure 2:**
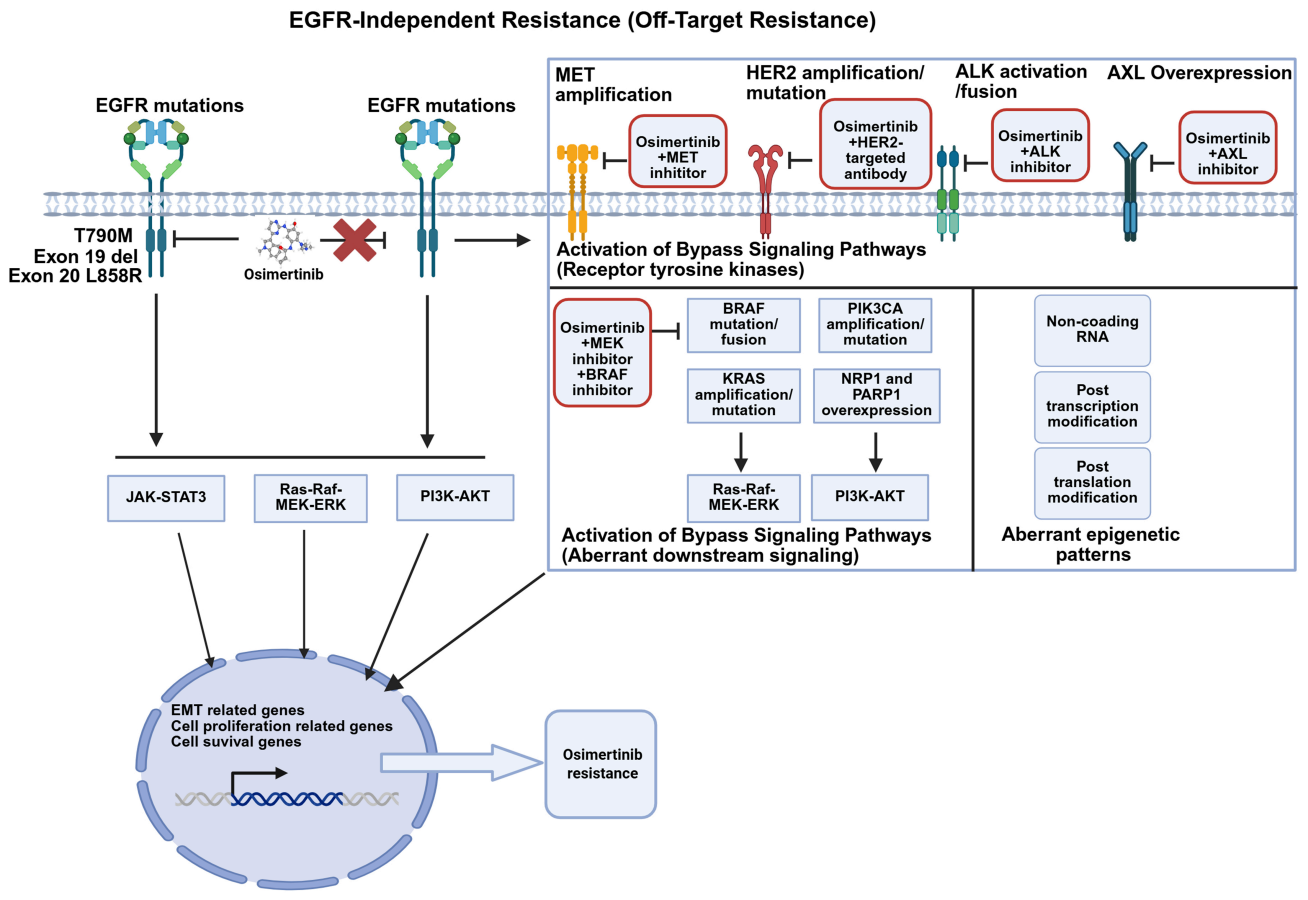
Alternative bypass signaling mechanisms of osimertinib-acquired resistance in EGFR-mutated NSCLC and corresponding management strategies. Transmembrane receptors such as MET, HER2, and AXL, as well as downstream pathways including PI3K and RAS, may undergo mutation, fusion, or amplification, leading to resistance to osimertinib (Created with BioRender.com. https://BioRender.com/wr8kb92). EGFR: Epidermal growth factor receptor; MET: mesenchymal-epithelial transition factor; HER2: human epidermal growth factor receptor 2; AXL: AXL receptor tyrosine kinase; ALK: anaplastic lymphoma kinase; PI3K: phosphatidylinositol 3-kinase; AKT: protein kinase B; RAS: rat sarcoma viral oncogene homolog; RAF: rapidly accelerated fibrosarcoma; MEK: mitogen-activated protein kinase kinase; ERK: extracellular signal-regulated kinase; JAK: Janus kinase; STAT3: signal transducer and activator of transcription 3; EMT: epithelial-mesenchymal transition; NSCLC: non-small-cell lung cancer.

**Figure 3:**
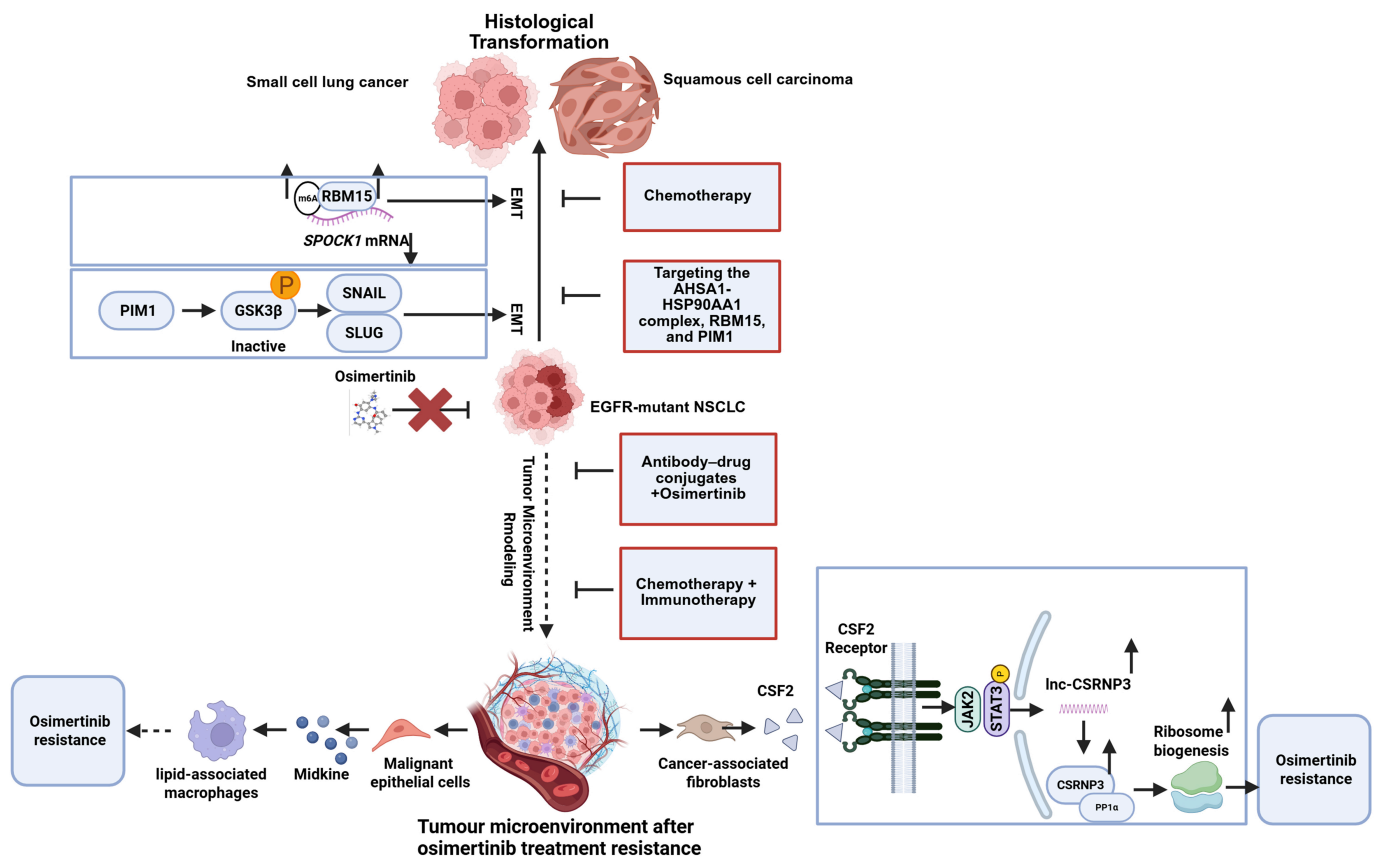
EMT and histological transformation mechanisms of osimertinib-acquired resistance in EGFR-mutant NSCLC and management strategies to overcome them. Cancer cells may undergo EMT or transform into SCC or SCLC (Created with BioRender.com. https://BioRender.com/bzr4mlu). EGFR: Epidermal growth factor receptor; NSCLC: non-small-cell lung cancer; EMT: epithelial-mesenchymal transition; SCC: squamous cell carcinoma; SCLC: small cell lung cancer; lncRNA: long noncoding RNA.

Acquired resistance mechanisms to osimertinib can be categorized into three main types: On-target resistance (EGFR-dependent pathway) [Figure 1], activation of Bypass Signaling Pathways [Figure 2] (EGFR-independent pathway), and histological transformation [Figure 3], as well as other unknown mechanisms. Table 1 summarizes the frequencies and detection methods of acquired resistance mechanisms.

**Table 1:** Osimertinib-acquired resistance mechanisms in EGFR-mutant NSCLC

**Resistance category**	**Key alteration**	**Representative frequency**	**Line of therapy where described**	**Location identified**
EGFR-dependent	C797S/C797X mutation (disrupts covalent binding)	7%-26%	Both first- and second-line	Patient ctDNA and tumor biopsies^[39-41]^
	EGFR rare mutation C796S, L718Q, L792X, L692V, L798I, L844V (reduce drug affinity)	< 5%	Both first- and second-line	Patient cfDNA and tumor biopsies^[39-44]^
	EGFR amplification (enhances activation of EGFR signaling)	15%-19%	Both first-line and second-line settings	Patient ctDNA and tumor biopsies^[39-41]^
EGFR-Independent	MET overexpression	5%-50%	Both first-line and second-line settings	Patient plasma/tissue^[39-41,46,63]^
	MET amplification (activates bypass pathways)	7%-17%	Both first-line and second-line settings	Patient plasma/tissue^[39-41]^
	HER2 gene amplification/mutation, ALK activation/fusion, BRAF mutation/fusion, KRAS amplification/mutation, PIK3CA amplification/mutation, FGFR fusion amplification/amplification fusion, and cell-cycle-related genes (activation of alternative signaling)	1.5%-11%	Any line	Patient cfDNA^[39,44,88]^
	NRP1 and PARP1 overexpression (activate the PI3K/AKT pathway)	Not quantified	No	PC9-ER cell line^[89,90]^
	MUC1-C and AP-1 overexpression (upregulate the EGFR expression)	Not quantified	No	H1975-OR- cell line^[91,92]^
	Noncoding RNA (miR-130a-3p, lncRNA LRTOR, LINC01559, circMYBL1)	Not quantified	No	Patient tissues and OR cell lines^[93-96]^
	Post-transcriptional and post-translational modifications (deNG IL-6, METTL14)	Not quantified	No	resistance of NSCLC cells^[97,98]^
Histologic transformation	SCLC and SCC transformation	2%-15%	Both first-line and second-line settings	Tissue biopsies^[39-41]^
	EMT	Not quantified	Pre-clinical and case reports; any line	Tissue biopsies^[39-41]^
	AHSA1-HSP90AA1 complex, RBM15, and PIM1 (promote the EMT)	Not quantified	No	Osimertinib-resistant NSCLC cells^[104-106]^

EGFR: Epidermal growth factor receptor; NSCLC: non-small-cell lung cancer; ctDNA: circulating tumor DNA; cfDNA: cell-free DNA; MET: mesenchymal-epithelial transition factor; HER2: human epidermal growth factor receptor 2; ALK: anaplastic lymphoma kinase; FGFR: fibroblast growth factor receptor; NRP1: neuropilin 1; PARP1: poly(ADP-ribose) polymerase 1; MUC1-C: mucin 1 C-terminal subunit; AP-1: activator protein 1; PI3K/AKT: phosphatidylinositol 3-kinase/protein kinase B; miR: microRNA; lncRNA: long noncoding RNA; circRNA: circular RNA; IL-6: interleukin-6; SCLC: small cell lung cancer; SCC: squamous cell carcinoma; EMT: epithelial-mesenchymal transition; AHSA1: activator of HSP90 ATPase activity 1; HSP90AA1: heat shock protein 90 alpha family class A member 1; OR: osimertinib-resistant.

### On-target (EGFR-dependent) resistance

#### EGFR secondary mutant

Osimertinib exerts its inhibitory effect on EGFR primarily by forming a covalent bond with the cysteine-797 residue located within the adenosine triphosphate (ATP)-binding pocket of EGFR exon 20. This mechanism allows it to irreversibly inhibit both sensitive EGFR mutations (including T790M) and C797X mutations. As a result, the C797 site has emerged as a susceptible locus for osimertinib resistance^[36,37]^. The substitution of cysteine (Cys) at position 797 with another amino acid disrupts the covalent bonding, conferring resistance to osimertinib^[38]^. The C797X mutation, with C797S being the “flagship” subtype, has become a key “gatekeeper” of acquired resistance to osimertinib. C797S mutations occur in approximately 7%-26% of patients who develop resistance after first-line or second-line osimertinib therapy^[39-41]^. In addition to the C797S mutation, other less common mutations such as C796S, L718Q, C797G, L792X L692V, L798I, and L844V have also been reported^[39,42-44]^ [Figure 1].

#### EGFR amplification

EGFR amplification has emerged as a frequent mechanism of acquired resistance to osimertinib, bypassing therapeutic inhibition by enhancing EGFR signaling pathway activation^[45]^. In relevant studies, EGFR amplification has been observed in approximately 7% of patients who developed resistance following first-line and second-line osimertinib treatment^[46,47]^.

#### Management strategies for on-target resistance

Patients who have developed resistance to osimertinib have been confirmed to harbor secondary EGFR mutations or amplification of EGFR gene by NGS or histological assays of tissue biopsies. Therefore, fourth-generation EGFR-TKIs - currently under development - should be prioritized, or alternative therapeutic options, including chemotherapy, should be considered. These Fourth-generation agents include ATP-competitive inhibitors and allosteric inhibitors. Allosteric inhibitors—such as the EGFR allosteric inhibitor 045J (EAI045J) and the allosteric, orally active EGFR inhibitor JBJ-04-125-02—bind to the inactive conformation of EGFR and block its activity, thereby inhibiting the proliferation of cell lines and tumor growth in preclinical models with L858R/T790M- and L858R/T790M/C797S-mutated EGFR, either alone or in combination with an EGFR antibody^[48-50]^. BLU-945, an inhibitor targeting EGFR+/T790M and EGFR+/T790M/C797S resistance mutations, is currently undergoing phase I/II clinical trials for the treatment of EGFR-driven NSCLC that has acquired resistance^[51,52]^.

The TKI silevertinib (BDTX-1535) has been shown to mitigate resistance to osimertinib^[53]^. Supporting this, the drug exhibits promising preliminary clinical activity in a phase I/II trial involving patients with EGFR mutations that confer acquired resistance to osimertinib^[54]^. Additionally, two EGFR mutant-selective inhibitors HS-10375 and BPI-361175 are currently under clinical investigation^[55]^. However, ATP-competitive and allosteric inhibitors, which share mechanisms of action with previous generations of drugs, offer the potential advantage of reducing the risk of on-target resistance. Nevertheless, on-target resistance remains a common issue for competitive inhibitors. Therefore, further investigation is warranted into strategies involving fourth-generation EGFR-TKIs or combinations such as gefitinib and osimertinib for “dual EGFR blockade”^[56]^. Moreover, recent studies have reported that the EGFR T790M/C797S mutant appears to upregulate pyruvate dehydrogenase kinase (PDK1)-driven glycolysis via the EGFR/protein kinase B-alpha (AKT1)/hypoxia-inducible factor 1α (HIF-1α) pathway. The combination of osimertinib and the PDK1 inhibitor has been shown to successfully overcome osimertinib resistance^[57]^, thus providing new insights for overcoming EGFR-dependent resistance.

However, the inhibitors described above target only known EGFR-dependent resistance mutations - principally by binding to the ATP-binding pocket - rendering them potentially ineffective against rare or uncharacterized mutations. Lung cancer organoid technology now allows systematic generation of patient-derived organoids carrying diverse EGFR mutations, including those emerging before or after osimertinib resistance^[58]^. The clustered regularly interspaced short palindromic repeats (CRISPR)/CRISPR associated protein 9 (Cas9)-mediated gene editing further expands this platform to encompass putative or yet uncharacterized EGFR alterations^[59]^. Coupled with advanced automated microfluidic chips, these organoids enable high-throughput drug screening^[60,61]^, providing a robust pre-clinical pipeline for precision therapy against EGFR-driven resistance.

### EGFR-independent resistance

#### Activation of bypass signaling pathways (RTKs)

Activation of bypass signaling pathways involves mutations in non-EGFR RTKs and disruptions in downstream pathways. Among these, MET amplification is the most prevalent non-EGFR RTK alteration that drives resistance to osimertinib^[46]^. In NSCLC, the “triple-threat” activation pattern of the MET pathway-gene amplification, protein overexpression, and exon 14 skipping mutations collectively represents its most clinically significant alteration profile^[62]^. When osimertinib inhibits the EGFR signaling pathway, acquired MET amplification establishes a bypass signaling route that activates the downstream phosphatidylinositol 3 kinase (PI3K)-protein kinase B (AKT) pathway^[39]^. In NSCLC, Mesenchymal-Epithelial Transition factor (c-Met/MET) overexpression is positively associated with the presence of EGFR mutations. Among advanced EGFR-mutant patients receiving osimertinib therapy, the prevalence of C-Met protein overexpression has been reported to range from 30.4% to 37.0%^[63]^.

Consequently, dual targeting of MET and EGFR has emerged as a valid strategy to combat resistance to EGFR osimertinib^[64,65]^. The combination of osimertinib with MET small-molecule inhibitor has been reported in both real-world pathological cases and clinical studies^[66]^. MET inhibitors, such as savolitinib, capmatinib, tepotinib, and crizotinib, have yielded tangible clinical outcomes in osimertinib-resistant NSCLC patients harboring MET amplification in TATTON (NCT02143466), SAVANNAH (NCT03778229), and INSIGHT2 (NCT03940703)^[67-69]^. However, the combination of Tepotinib or Crizotinib with osimertinib did not show clinical benefit in patients with osimertinib-resistant NSCLC harboring MET exon 14 skipping mutations^[70,71]^. In contrast, Capmatinib in combination with osimertinib demonstrated clinical benefit in osimertinib-resistant NSCLC patients with MET alterations^[72]^. On the other hand, Amivantamab, a bispecific antibody targeting MET and EGFR, has been approved by the U.S. Food and Drug Administration (FDA) for the first-line setting and for the treatment of NSCLC patients with one of the two common EGFR mutations who have progressed on osimertinib^[73]^. Amivantamab combination with Lazertinib has demonstrated efficacy in patients who had progressed on osimertinib and platinum-based chemotherapy in CHRYSALIS-2 trial (NCT04077463)^[74]^. Additionally, the MET-antibody-drug conjugate (ADC) antibody, Teliso-v, has shown antitumor activity in tumors with MET overexpression or amplification. Teliso-v in combination with osimertinib exhibited robust efficacy alongside a manageable safety profile in patients with EGFR-mutated advanced/metastatic NSCLC harboring c-Met overexpression in a Phase I/Ib trial^[75,76]^. However, owing to the marked heterogeneity of MET amplification, the response to MET inhibitors differs among patients with divergent gene copy numbers (GCNs)^[77]^. To date, most therapeutic investigations have been confined to phase I/II trials in which comparative efficacy and safety have remained inconclusive. Consequently, large-scale randomized controlled trials, harmonized biomarker-driven enrollment criteria, and refined dosing/scheduling of combination regimens are urgently warranted to achieve robust and reproducible clinical benefit.

Besides MET amplification, human epidermal growth factor receptor 2 (HER2) gene amplification or mutation^[78]^ and anaplastic lymphoma kinase (ALK) activation/fusion^[79]^ are also common non-EGFR RTK alterations. Trastuzumab deruxtecan (T-DXd), a HER2-targeting antibody-drug conjugate in combination with osimertinib, is currently under clinical investigation for NSCLC patients harboring HER2 overexpression or amplification following osimertinib treatment. The ORR was 4%, with a median PFS of 2.8 months^[80]^. Case reports have demonstrated that fama-trastuzumab-deruxtecan can be safely combined with osimertinib to treat patients with HER2 resistance mutations who progress after osimertinib treatment, with a PFS of eight months in such patients^[81]^. Moreover, combination therapies with ALK inhibitors have entered phase III clinical trials and have demonstrated efficacy^[82]^. Targeting the resistance mechanism of AXL receptor tyrosine kinase overexpression, the combination of an AXL inhibitor (Anlotinib) and osimertinib has been employed to enhance antitumor effects by inhibiting the c-MET/MYC/AXL axis, thereby reversing osimertinib resistance in NSCLC^[83]^. Furthermore, a dual-targeted antibody (mAb654) against EGFR and AXL in combination with osimertinib has been shown to delay resistance and warrants further clinical investigation^[84]^. AXL inhibitors can also be delivered via a nanovesicle delivery system - associated with the GSH-AXL axis - to restore osimertinib sensitivity^[85,86]^. Notably, a triple therapy comprising osimertinib, an AXL inhibitor, and an FGFR inhibitor has demonstrated robust efficacy in EGFR-mutated NSCLC patients^[87]^. Despite the clinical benefits and favorable safety profiles, the efficacy of these approaches remains limited.

#### Activation of bypass signaling pathways (aberrant downstream signaling)

Numerous studies have confirmed that rapidly accelerated fibrosarcoma B-type (BRAF) mutation/fusion, Kirsten rat sarcoma viral oncogene homologue (KRAS) amplification/mutation, phosphatidylinositol-4,5-bisphosphate 3-kinase catalytic subunit alpha (PIK3CA) amplification/mutation, FGFR fusion/amplification, and aberrant alterations in cell-cycle-related genes constitute key acquired resistance mechanisms following osimertinib treatment^[39,44]^ [Figure 2]. These mechanisms are beyond the scope of this discussion.

Meanwhile, efforts to overcome osimertinib resistance by targeting other oncogenic alterations continue to expand. The primary approach involves combining osimertinib with inhibitors targeting specific resistance-associated genetic alterations. This combination represents a promising therapeutic option for osimertinib-resistant NSCLC. For instance, in patients harboring acquired BRAF mutations, a clinical trial has evaluated a triple regimen comprising osimertinib, Trametinib (BRAF inhibitor) and Dabrafenib (MEK inhibitor)^[88]^.

Further research is warranted to develop novel drugs and innovative therapeutic strategies against EGFR-TKI resistance driven by these aberrant genes. Notably, in the past two years, researchers have also discovered that overexpression of neuropilin 1 (NRP1) and poly(ADP-Ribose) polymerase 1 (PARP1) has been shown to activate the PI3K/AKT pathway^[89,90]^; Activation of MUC1-C and AP-1 promotes epithelial-mesenchymal transition (EMT) via the EGFR/ERK/AKT axis^[91,92]^. These findings underscore their potential as therapeutic targets to reverse osimertinib resistance.

#### Noncoding RNA

Noncoding RNAs can also drive osimertinib resistance in NSCLC. For instance, miR-130a-3p within extracellular vesicles regulates osimertinib resistance in lung adenocarcinoma by targeting dwarf-related transcription factor 3. Overcoming resistance to EGFR TKIs is crucial for improving treatment outcomes in lung cancer^[93]^. The long noncoding RNA (lncRNA) LRTOR contributes to acquired osimertinib resistance in NSCLC by promoting a Yes-associated protein (YAP)-positive feedback loop^[94]^. LINC01559 promotes osimertinib resistance in NSCLC by acting as a competing endogenous RNA (ceRNA) to regulate the miR-320a/IGF2BP3 axis^[95]^. circMYBL1 (has_circ_0136924) is downregulated following osimertinib resistance^[96]^. These noncoding RNAs also represent promising therapeutic and prognostic targets with potential for overcoming osimertinib acquired resistance in EGFR-mutant-positive NSCLC.

#### Post-transcriptional and post-translational modifications

Post-transcriptional and post-translational modifications also contribute to osimertinib resistance in NSCLC. For instance, N-glycosylation-defective (deNG) interleukin (IL)-6 (the deglycosylated state of IL-6) has been shown to promote resistance in NSCLC^[97]^. Methyltransferase 14, N6-adenosine-methyltransferase non-catalytic subunit (METTL 14)-mediated N6-methyladenosine (m6A) modification of Bim messenger RNA (mRNA) enhances the sensitivity of EGFR-mutated NSCLC cells to osimertinib^[98]^. These findings represent new directions for addressing osimertinib resistance in NSCLC.

### Histological transformation

After osimertinib resistance develops, histological transformation may occur, most commonly to squamous cell carcinoma (SCC) or small cell lung cancer (SCLC), both of which are associated with poor prognosis [Figure 3]^[99]^. There is evidence suggesting that the incidence of histological transformation in osimertinib-resistant NSCLC is approximately 2%-15%. SCLC is more common, representing 10%-15% of cases^[33]^. For patients with osimertinib-resistant, repeat biopsy should be performed, and treatment should be guided by precise subtype identification and emerging therapies. EGFR-TKI resistance caused by SCLC transformation is typically associated with an aggressive clinical course and poor prognosis, and is unresponsive to immunotherapy. Marcoux’s data indicate that SCLC-transformed cases are usually characterized by mutations in retinoblastoma 1 (Rb1), tumor protein p53 (TP53) and PIK3CA. Although these tumors frequently respond to platinum-etoposide or paclitaxel, they rarely respond to checkpoint inhibitors; the underlying mechanisms remain to be elucidated^[100]^. When mutations in the TP53 and RB1 genes are present, the combination of osimertinib and chemotherapy can delay the transformation of cancer cells. Clinical trial results show that 82% of patients experienced a significant reduction in tumor lesions^[101]^. For patients who undergo SCLC transformation following osimertinib resistance, standard chemotherapy with etoposide plus a platinum agent is recommended. Although response rates are generally high, remission duration is limited; a retrospective study reported an ORR of 54% and a median OS of 10.9 months^[100]^. The addition of Anlotinib to this regimen increases the median OS to 14 months^[102]^. Following relapse after chemotherapy, Tarlatamab - a bispecific delta-like ligand 3 (DLL3) × cluster of differentiation 3 (CD3) T-cell engager that forms an immunologic synapse and activates T-cell cytotoxicity - has been approved in the United States for small-cell lung cancer^[103]^. This milestone provides the first clinical evidence supporting immunotherapy in SCLC-transformed tumors and highlights the urgency for expanded investigation. In patients with SCC transformation, platinum-based doublet chemotherapy remains the first-line standard for advanced lung SCC. Programmed cell death ligand 1 (PD-L1) testing of the transformed tissue should be considered to guide chemo-immunotherapy decisions, although definitive evidence is lacking; median OS is approximately 11 months and prognosis remains poor.

The primary mechanisms include EMT, which can be a precursor to or occur concomitantly with histological transformation. EMT also promotes cell dedifferentiation and provides a basis for histological transformation. Recent studies have found that proviral integration site for Moloney murine leukemia virus 1 (PIM1) kinase promotes EMT-related osimertinib resistance in EGFR-mutated NSCLC by modulating the glycogen synthase kinase-3β (GSK3β) signaling pathway. PIM1 kinase acts as a driver of osimertinib-resistant NSCLC cells associated with EMT^[104]^. RNA binding motif protein 15 (RBM15) enhances m6A modification of CWCV and Kazal-like domains proteoglycan 1 (SPOCK1) mRNA, thereby upregulating EMT-mediated acquired osimertinib resistance via a bypass signaling pathway^[105]^. Under osimertinib stress, the ATPase HSP90 family regulator 1–heat shock protein 90 alpha family class A member 1 complex (AHSA1–HSP90AA1) stabilizes interferon-induced protein 6 (IFI6) and transforming growth factor beta 1 (TGFB1), promoting protein kinase B (AKT) phosphorylation and epithelial-to-mesenchymal transition (EMT), thereby exacerbating resistance^[106]^. In 2025, researchers combined a biobank of patient-derived EGFR-mutant lung cancer organoids (ELCOL) with single-cell RNA-seq and complementary multi-omic analyses to delineate the mechanisms driving histologic transformation. They identified cyclin-dependent kinases 4 and 6‌ (CDK4/6) inhibition as an effective strategy to overcome this resistance mode^[107]^. These findings suggest that the AHSA1-HSP90AA1 complex, RBM15, and PIM1 represent potential biomarkers and therapeutic targets for overcoming primary EGFR-TKI resistance, complementing standard chemotherapy with etoposide and platinum agents.

### Unknown resistance mechanisms

Despite intensive global efforts to delineate resistance mechanisms, repeat tissue and/or liquid biopsies analyzed by integrated multi-omic NGS - including proteomics, spatial transcriptomics, and assay for transposase-accessible chromatin with high-throughput sequencing (ATAC-seq) - fail to identify known resistance mutations or amplifications in 30%-50% of patients progressing on osimertinib, indicating that undefined mechanisms persist. Functional validation of candidate variants emerging from these analyses can be performed in patient-derived organoids or xenograft models to establish their role in resistance, thereby informing trial enrollment and subsequent therapy for later-line patients. Such mechanisms may involve the tumor microenvironment, immune evasion, and unknown genetic mutations or epigenetic regulation.

A case report described a stage IVa (T3N3M1a) lung adenocarcinoma with multiple intrapulmonary metastases harboring EGFR Ex19del. Following disease progression on osimertinib, in the absence of detectable resistance mechanisms (T790M, MET amplification, KRAS mutation, or SCLC transformation) and within an immunosuppressed microenvironment, the patient was subsequently treated with antigen-specific cytotoxic T-lymphocyte (ACTL) therapy. This intervention led to a complete clinical response (CCR), which has been sustained for six years. This illustrates ACTL’s potential to remodel the tumor immune milieu and overcome osimertinib resistance of undefined etiology, in contrast to immune-checkpoint inhibitors, which show limited activity in EGFR-mutant advanced NSCLC but confer clear benefit in EGFR-wild-type metastatic disease^[108]^. Integrated proteomic, spatial transcriptomic and ATAC-seq data revealed that AT-rich interaction domain 1A (ARID1A) loss activates the enhancer of zeste homolog 2/phosphatase and tensin homolog/E2F transcription factor 1 (EZH2/PTEN/E2F1) axis, suppressing programmed cell death, upregulating PD-L1 expression and promoting its nuclear localization. Consequently, the ras-GEF domain-containing family member 1A (RASGEF1A) promoter is activated, engaging Ras signaling and driving osimertinib resistance. *In vivo*, lipid-nanoparticle-delivered PD-L1 small interfering RNA (siRNA) reversed these ARID1A-knockdown-induced changes and restored osimertinib sensitivity^[109]^. A chemo-immunotherapy backbone may be considered for patients with undefined resistance mechanisms; the choice of immunotherapeutic component should be guided by post-progression PD-L1, NADPH oxidase 4‌ (NOX4)/interleukin-8 (IL-8) profiling, with concurrent monitoring of pulmonary function and systemic inflammatory markers to minimize toxicity.

Additionally, researchers revealed that cancer-associated fibroblasts (CAFs) from osimertinib-resistant lung adenocarcinoma (LUAD) tissues produce higher levels of colony-stimulating factor 2 (CSF2) compared to those from osimertinib-sensitive tissues. This increased CSF2 production activates the Janus kinase 2 (JAK2)/signal transducer and activator of transcription 3 (STAT3) signaling pathway in LUAD cells, leading to upregulation of long noncoding RNA - cysteine-serine-rich nuclear protein 3 (lnc-CSRNP3). Consequently, chromodomain helicase DNA binding protein 9 (CHD9) is recruited to promote expression of the nearby gene CSRNP3, thereby inhibiting protein phosphatase 1 catalytic subunit alpha (PP1a) phosphatase activity, and ultimately inducing osimertinib resistance via enhanced ribosome biogenesis^[110,111]^. Moreover, a specific type of lipid-associated macrophage population, Ribonuclease A Family Member 1 (RNASE1-M), which is regulated by Midkine‌ (MDK), has been identified as being associated with osimertinib resistance and lung cancer development. MDK levels are significantly elevated in the cerebrospinal fluid and plasma of patients with leptomeningeal metastasis (LM). Malignant epithelial cells in the cerebrospinal fluid may evade immune surveillance by engaging the cluster of differentiation 47 (CD47) - signal regulatory protein α (SIRPA) axis with RNASE1-M, promoting their polarization toward a macrophage M2 (M2)-like phenotype characterized by lipid metabolism and phagocytic dysfunction^[112]^. Collectively, these findings underscore the potential of immune microenvironment modulation as a therapeutic strategy to overcome osimertinib resistance in lung cancer. They also highlight the urgent need to reassess the optimal treatment approach - particularly whether combined immunotherapy or conventional chemotherapy is more effective in this context.

For patients with undefined resistance mechanisms, a broad-spectrum strategy combining ADCs with osimertinib may be employed. For example, the trophoblast cell surface antigen 2‌ (TROP2)-targeted ADC (Dato-DXd) combined with osimertinib demonstrated promising outcomes, achieving a median PFS of 9.5-11.7 months - outperforming conventional chemotherapy^[113-115]^.

Alternatively, a chemotherapy-immunotherapy combination represents another viable option. In the multicenter, open-label, randomized phase II OptiTROP-Lung03 trial (NCT05631262), the TROP2-directed ADC sacituzumab tirumotecan was evaluated in patients with advanced EGFR-mutant NSCLC who had progressed on osimertinib and platinum-based chemotherapy but had no identifiable resistance mechanism. The ORR was 45% *vs.* 16% with docetaxel (*P* < 0.001), with median PFS of 6.9 months *vs.* 2.8 months, corresponding to a 70% reduction in progression or death risk [hazard ratio (HR) 0.30; 95% confidence interval (CI): 0.19-0.47]. A prespecified interim analysis revealed a 51% reduction in mortality risk (HR 0.49; 95%CI: 0.31-0.76). Sacituzumab tirumotecan exhibited a manageable safety profile with no new signals, leading to its approval as the first TROP2-targeted ADC for lung cancer globally, establishing a new standard of care and ushering in an era of precision-targeted chemotherapy. This regimen offers meaningful survival benefit and improved quality of life, advancing the field toward precision, personalization, and long-term survival^[116]^.

## CONCLUSION AND PROSPECT

The mechanisms of osimertinib acquired resistance are highly complex and heterogeneous, most likely attributable to intratumoral heterogeneity. Once resistance emerges, subsequent treatment should be tailored to the molecular resistance subtype [Table 1]. This review provides a comprehensive analysis of the mechanisms underlying osimertinib resistance in EGFR-mutant NSCLC. Although mechanism-matched strategies have been proposed, most remain in phase I/II trials or pre-clinical models. Supplementary Table 1 summarizes the clinical and experimental data of EGFR-mutant NSCLC patients who developed resistance to osimertinib. EGFR secondary mutations, MET amplification and histologic transformation account for the majority of osimertinib acquired resistance, with EGFR-directed regimens being the most mature. A sizeable proportion of resistant tumors, however, are driven by undefined mechanisms such as immune-microenvironmental rewiring consequent to altered cellular composition, and the clinical efficacy of corresponding therapies remains highly variable.

Current research aims to counter osimertinib resistance by targeting novel pathways, including NRP1^[89]^, PARP1^[90]^, MUC1-C^[91]^, heat shock protein 90 (HSP90) signaling pathway^[106]^, branched chain aminotransferase 1 (BCAT1)^[117]^, epigenetic^[98]^ and noncoding RNAs^[89,95]^, and by deploying inhibitors against specific resistance mutations (e.g., MET inhibitors^[67]^, AXL inhibitors^[118]^ and Antibody drug conjugates (ADCs)^[119]^)in combination with osimertinib [Supplementary Table 1]. Additionally, the use of osimertinib as adjuvant therapy in operable EGFR-mutated NSCLC^[28]^, as neoadjuvant therapy in advanced EGFR-mutated NSCLC^[27]^, and in combination therapies^[120]^ may induce greater heterogeneity and more complex resistance mechanisms. Therefore, single-cell spatiotemporal multi-omics should be leveraged to dissect how the tumor microenvironment, immune-evasion programs and mutation-specific genetic, transcriptomic, epigenetic and post-translational signatures (e.g., phosphorylation, ubiquitination) collectively drive heterogeneity of osimertinib acquired resistance. Integration of these datasets with patient-derived organoids and CRISPR-based editing will accelerate validation of newly identified resistance drivers or pathways.

In the future, real-world data [such as the FLOURISH study (NCT04391283‌)]^[121,122]^, along with dynamic monitoring technologies, will provide important evidence for clinical decision-making. Further integration of multi-omics data is required to achieve precision medicine throughout the entire treatment process. Optimization of combination therapies, including those with chemotherapy and immune checkpoint inhibitors [e.g., cytotoxic T-lymphocyte-associated protein 4 (CTLA4) blockade]^[123]^, is also essential to balance efficacy and toxicity. The development of novel drugs, including bispecific antibodies, nanovesicle delivery systems^[86]^ and inhibitors targeting epigenetic regulation, represents promising strategies. Additionally, some repurposed drugs may also help overcome osimertinib resistance. For instance, tanshinone IIA can reverse osimertinib resistance by inhibiting lipid synthesis mediated through the sterol regulatory element-binding protein (SREBP) pathway *in vitro* and *in vivo*^[124]^. Similarly, ethyl caffeate, a traditional Chinese medicine component, can delay resistance by downregulating MET and modulating the AKT/PI3K/PTEN pathway^[125]^. These findings offer potential therapeutic options that aim to improve outcomes for osimertinib-resistant EGFR-mutant NSCLC patients.

It is, however, noteworthy that the expanding use of fourth-generation EGFR-TKIs or combination regimens is likely to select for novel resistance clones. These may include newly arising EGFR mutations that reduce drug affinity, structural alterations outside the ATP-binding pocket that impair inhibitor binding, or EGFR amplification-driven activation of bypass pathways. By integrating artificial-intelligence prediction of probable resistance alleles with CRISPR-based engineering in patient-derived lung cancer organoids and automated screening platforms, we can generate large isogenic mutant libraries for functional annotation and high-throughput drug testing, thereby prospectively identifying emergent resistance hotspots and candidate next-generation inhibitors. Nevertheless, organoid-only models remain inadequate for dissecting immune-microenvironment-mediated osimertinib resistance; microphysiological systems that co-culture lung cancer organoids with immune components will be required to interrogate such mechanisms and screen corresponding therapeutic strategies. With the advancement of artificial intelligence, it has become feasible to fully utilize dynamic genomic testing and AI-based models to predict resistance pathways and formulate precise treatment plans, thereby promoting individualized therapeutic strategies. As our decoding of the biological mechanisms underlying osimertinib acquired resistance and clinical datasets that guide post-resistance treatment options continue to accumulate, further improvements in treatment will provide more precise and personalized options, ultimately enhancing clinical outcomes for patients with osimertinib-resistant disease.

## DECLARATIONS

### Copyright

© The Author(s) 2025.
